# *WTX* R353X mutation in a family with osteopathia striata and cranial sclerosis (OS-CS): case report and literature review of the disease clinical, genetic and radiological features

**DOI:** 10.1186/1824-7288-38-27

**Published:** 2012-06-20

**Authors:** Anna Maria Zicari, Luigi Tarani, Daniela Perotti, Laura Papetti, Francesco Nicita, Natascia Liberati, Alberto Spalice, Guglielmo Salvatori, Federica Guaraldi, Marzia Duse

**Affiliations:** 1 Department of Pediatrics, Policlinico Umberto I, Sapienza University, Rome, Italy; 2 Department of Preventive and Predictive Medicine, Fondazione IRCCS Istituto Nazionale dei Tumori, Milan, Italy; 3 NICU- Department of Medical and Surgical Neonatology, IRCCS Bambino Gesu’ Children’s Hospital, Rome, Italy; 4 Division of Endocrinology, Diabetology and Metabolism, Department of Internal Medicine, S. Giovanni Battista Hospital, University of Turin, Corso Dogliotti, 14, 10126 Turin, Italy

**Keywords:** Osteopathia striata, Cranial sclerosis, Horan-Beighton syndrome, WTX, Bone dysplasia

## Abstract

Osteopathia striata with cranial sclerosis (OS-CS) or Horan-Beighton syndrome is a rare X-linked dominant inherited bone dysplasia, characterized by longitudinal striations of long bones and cranial sclerosis. Patients can be asymptomatic or present with typical facial dysmorphism, sensory defects, internal organs anomalies, growth and mental retardation, depending on the severity of the disease. *WTX* gene (Xq11) has been recently identified as the disease causing gene. Aim of this article is to present the case of a 6 year old girl initially evaluated for bilateral hearing loss. Patient’s head CT scan pointed out sclerosis of skull base and mastoid cells, and abnormal middle-ear ossification. Clinical examination of the patient and her mother were suspicious for OS-CS. The diagnosis was confirmed by X-rays examination showing typical longitudinal striation. Genetic analysis allowed the identification of maternally transmitted heterozygous nonsense c.1057C>T (p.R353X) *WTX* gene mutation. We also provide a systematic review of currently available knowledge about clinical, radiologic and genetic features typical of the OS-CS.

## Introduction

Osteopathia striata with cranial sclerosis (OS-CS; OMIM#300373) is a rare, X-linked dominant inherited skeletal dysplasia (prevalence 0.1/1 million people [1]), part of sclerosing bone dysplasias [2-4], a group of disorders characterized by abnormally dense bones as a result of the prevalence of bone formation by osteoblasts over bone reabsorption by osteoclasts. Striated methaphyses were firstly described by Voorhoeve in 1924 [5]; thirty years later, Hurt [3] reported a case of association with cranial sclerosis. To date, about one hundred cases have been reported. Age at diagnosis and clinical presentation is highly variable, ranging from asymptomatic to neonatal lethal cases. Skull thickening, responsible for characteristic facies, and linear striations in the metaphyseal region of the long bones and pelvis represent disease main features, the latter representing a key feature for differential diagnosis [2-4]. Nervous and internal organs defects can be associated. Causing-disease mutations of *WTX* (Wilms Tumor in the X; also called *FAM123B* and *AMER1*) have recently been identified.

Aim of this paper is to present the first case of OS-CS with maternal transmission of a heterozygous 1057C>T *WTX* gene mutation, and to summarize currently available knowledge on OS-CS clinical, radiological and genetic features.

## Case report

**Figure 1:**
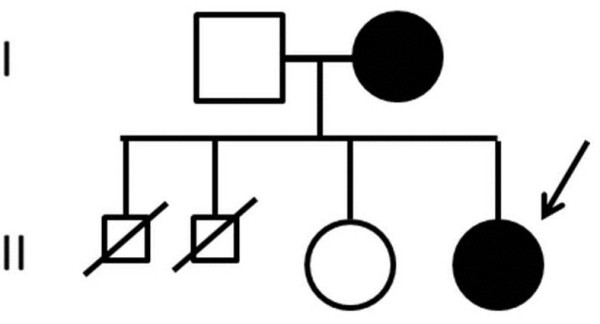
Family tree showing the affected patient (II.4; black arrow) and her affected mother (I.2), the two male aborted fetuses (II.1, II.2) and healthy sister (II.3).

**Figure 2:**
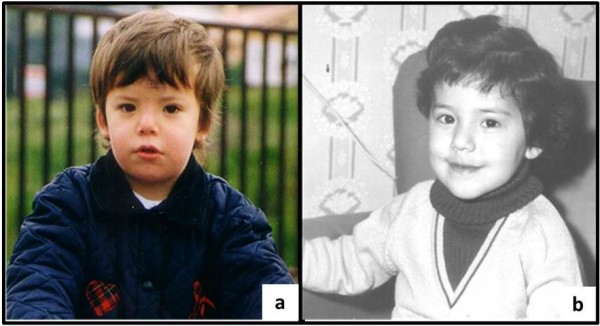
**Picture of the patient at age 2 (a) showing the typical narrow forehead with frontal bossing, hypertelorism with epicanthal folds, up-slanting palpebral fissures, wide depressed nasal bridge, mid macrocephaly**. Picture of the patient’s mother at age 3 (b) showing mild hypertelorism with epicanthal folds and a slightly depressed nasal bridge.

**Figure 3:**
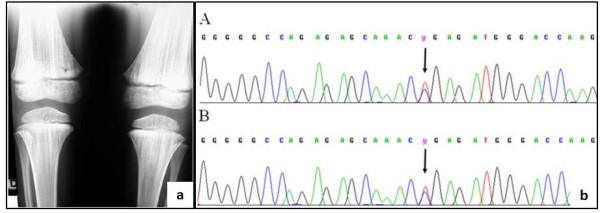
**X-ray of the patient’s femur showing OS-CS typical bone longitudinal striations (a)**. Sequence analysis of *WTX* gene performed on peripheral blood leukocytes in the patient (panel A) and her mother (panel B) showing the heterozygous nonsense mutation 1057C>T (arrows) (**b**).

The proband came to our attention at age 6 for evaluation of recurrent otitis media and bilateral hearing loss [6]. She was born at the 40th week of gestation after spontaneous vaginal delivery from unrelated, apparently healthy parents. Her weight at birth was 3.9 kg (>97th centile); no facial anomalies were noted. Her mother had previously had two male abortions and a healthy daughter (Figure 1). A skull X-ray, performed at age 3 because of high fever, right-eye and nasal bridge swelling, had shown dense basilar bones and decreased pneumatization of the mastoid and ethmoid cells [6]. Physical examination performed at age 6 showed narrow forehead with frontal bossing, hypertelorism with epicanthal folds, up-slanting palpebral fissures, wide depressed nasal bridge, mild macrocephaly (head circumference 53.8 cm, slightly >2SD), hypoplastic maxillae and delayed dentition, low-set ears (Figure 2a), and pectus excavatum. Inspection of the oral cavity revealed a high-arched palate and dental-position abnormalities. Otologic examination showed bilateral mild retraction of the tympanic membrane. The audiometric test revealed a severe mixed hearing loss with a wide gap in air conduction on the left side and medium-grade sensor neural hearing loss on the right side. High-resolution computed tomography (CT) of the temporal bone showed bilateral thickening and bone sclerosis of the skull base and mastoid cells with narrowing of the middle ear cavity, mastoid antrum, and eustachian canal, bilateral abnormal ossicular fixation to the bone surface of the middle ear cavity, and the presence of phlogistic tissue in the middle ear, confirmed by magnetic resonance [6]. X-rays of the skull and legs were performed because of clinical and CT findings suspicious for OS-CS, and revealed cranial vault’s sclerosis (more pronounced in frontal and parietal bones, and involving the orbits, sphenoid and petrous bones), and linear striations in the pelvis and metaphyseal region of long bones of lower and upper limbs (Figure 3a). Neurological examination excluded cranial nerves’ dysfunction and psychomotor delay. Cardiovascular and gastroenterological examinations were also normal.

Patient’s mother presented very similar facial features; particularly, she had mild hypertelorism with epicanthal folds, a slightly depressed nasal bridge and hypoplastic maxillae (Figure 2b). X-rays examination of lower limbs revealed longitudinal sclerotic striations in the metaphysis of femurs and tibias.

### Molecular analysis of WTX

The DNA from peripheral blood leukocytes of the patient and of her mother was extracted using QIAamp DNA Blood Mini kit (QIAGEN, Milan, Italy). Sequencing of the entire coding region of *WTX* was performed as previously described [7] and allowed the identification of the heterozygous nonsense mutation c.1057C>T, leading to a truncated protein (p.R353X) (Figure 3b). This mutation had been previously reported as a *de novo* mutation only in two sporadic cases [8].

## Literature review

A systematic literature review was performed using PubMed database entering the words “osteopathia striata”, “cranial sclerosis”, “Horan-Beighton syndrome”, “WTX gene”. Articles written in English and published since 1953 were included (Additional file 1).

## Clinical and radiological features (Additional file 1)

The clinical presentation is highly variable even within the same family. Patients can be asymptomatic and accidentally diagnosed during X-rays examinations, or present with disabling physical anomalies, sometimes leading to premature death. Age at diagnosis varies from neonatal period to the 5^th^ decade [9].

### Craniofacial dysmorphism

Skull thickening (cranial sclerosis) is the most typical and early feature [1,6,8,10-37] (85%), often presenting before longitudinal bone striations, and typically affects cranial vault, base and some facial bones, leading to sinuses obliteration and reduction of mastoid pneumatization [1,10,12-14,19,21,26,33,34] (sometimes responsible for failure to thrive). Facial dysmorphisms include macrocephaly (43%) [2,11-17,19,20,27-32,34,36-39], frontal and occipital bossing (32%) [2,11-15,17,19-21,30-33,36], mandible overgrowth with protuberance of the jaw and dental malocclusion (12%) [2,11,14,23] (giving a leonine appearance), ocular hypertelorism [6,8,10,14,19,20,23,27,29-31,38-40], down-slanting palpebral fissures [19,28,31], low set broad nasal bridge (29%) [1,2,6,8,12,14,15,17,18,20,23,30-32,37,38,41,42], narrow high-arched or cleft palate (Pierre Robin’s triad) (12%) [2,6,8,14-16,19,21,30,31,35] and low set dysplastic ears (9%) [6,12,19,20,29-31,33,39,42].

### Skeletal defects

Longitudinal striations can be typically demonstrated in long bones (73%), less frequently in pelvis (fan-like striations) (19%)[1,2,6,8,10-15,17-22,24-26,28-32,34-38,40,42], vertebrae and ribs, and can be associated to diffuse osteosclerosis (35%) [3,21,25,26,28-32,34,36,43]. Patients can also present thoracic and vertebral anomalies (2%) [8,13,14,19-21,28-32,35,42], digital flection contractures, phalangeal duplication, syndactyly, short or absent fibula, club feet (3%) [8,10,14,16,17,20,28-31,35,36,38,40].

### Neurological manifestations

Hearing loss (conductive or mixed type) is the most common neurological manifestation (46%) [1,2,6,8,11,14,16,17,19-23,27,28,30,31,33-38,40,41,43], resulting from narrowing of external auditory canal, impaired mobility of middle ear ossicles, damage to the inner ear or auditory nerve entrapment. Narrowed nerves’ canals and foramina are responsible for nerve encroachment and palsies [1,14,17,19,20,30,31,37-39,41,42]. A minority of patients presents intellectual impairment associated to defects of the central nervous system (i.e. ventricular dilatation, abnormal gyration, agenesis of corpus callosum) and developmental delay (overall, 20%) [2,8,14,17,19,20,29-33,35,37-40,43].

### Internal organs defects

Patients can finally present congenital heart defects (i.e. patent ductus arteriosus, atrial and ventricular septal defects, valvular and conductive defects) (11%) [8,11,12,14,20,27,29-31,35,37,41,43], respiratory (i.e. laryngotracheomalacia, respiratory distress, nasal obstruction, recurrent bronchitis) (13.5%) [6,13,17,19-21,23,28,30,31,37-39], gastrointestinal (i.e. malrotation, omphalocele, Hirschsprung disease, imperforate anus) (12%) [8,12,13,21,23,29-32,35] and urogenital (i.e. cryptorchidism, micropenis, duplicated ureter) (10%) [8,20,29-31,44] anomalies.

## OS-CS genetics and genetic-phenotype correlations

OS-CS has been classically considered an autosomal dominant condition with high penetrance and variable intra- and interfamilial expressivity [4,9,12,15-17,21,43] until the recent identification of mutated *WTX* (Wilms Tumor in the X; also called *FAM123B* and *AMER1*) within proximal Xq11.2 in affected families [8]. WTX can be spliced: the full length form encodes for a 1135 aminoacid protein, WTXs1, which possesses three binding sites for APC (adenomatous polyposis coli), a β-catenin- (C-terminal), a WT1- and a PIP2 (phosphatidylinositol 4.5 bisphosphate, at N-terminus)- binding site; and a shorter isoform, WTXs2 (missing aminoacids 50-326) keeps the first APC- binding domain [45-47]. WTX interacts with AXIN1, APC and β-TrCP2, promoting the ubiquitination and proteasomal degradation of β-catenin (binding site located at the C-terminal) [48] and the inhibition of the WNT signaling pathway [45]. Both isoforms can bind β-catenin but, while WTXs1 has a predominant plasma membrane and cytoplasmic localization, WTXs2 (missing PIP2-binding site) is retained in the nucleus, suggesting the importance of cellular localization for protein function [8,45,47]. In absence of WTX, β-catenin accumulates in the nucleus and works as a transcriptional co-activator [31]. Because WNT signal pathway is involved in various embryonic development (including commitment of mesenchymal progenitor cells and differentiation of osteoblast precursors [49]) and homeostasis of adult tissues [50], its enhanced activity, due to mutations of the suppressor *WTX,* is responsible for the different alterations and phenotypes observed in patients.

Hemizygous males usually are more severely affected than heterozygous females and present, other than more marked bone sclerosis, craniofacial abnormalities, gross structural malformations of bones and internal organs, and significant pre- and postnatal lethality [20]. More recently, families with mildly affected males, characterized by longer longevity, mild short stature and neurodevelopmental disability, have been reported [18,26,35,37,43], although the phenotype has not been completely defined yet [20]. A genotype-phenotype correlation, where patient with 5′ *WTX* mutations were more severely affected than patients with 3′ *WTX* mutations was proposed by Jenkins [8], but not confirmed by Perdu [30,31] who described mildly affected patients with 5′ WTX mutation, indicating a variable correlation between position of mutation and the severity of the phenotype. Because 5′ mutations produce a truncated, non-functioning protein, mild phenotype could be the result of a compensatory activity of WTXs2, even if the exact molecular mechanisms remain unidentified [20,46].

Metaphyseal striations can be seen only in females, and males with mosaic mutations [18,37,40]. A reasonable explanation is the differential lionization of osteoblasts for which, similarly to what happens in heterozygous females presenting with random X chromosome inactivation patterns, only a certain amount of osteoblasts is affected [33].

Somatic mutations and deletions of *WTX* have been reported in 6 to 30% of patients with Wilms tumors (WT), a kidney cancer typical of childhood, arising from multipotent mesenchymal kidney precursors [7,44,46,49]; *WTX* has later been identified as OS-CS disease-causing-gene, both in familial and sporadic cases. Despite germline mutations in tumor suppressor gene confer an elevated risk for cancer, and patients with WT and OS-CS share a similar distribution of WTX mutations, OS-CS is not associated with an increased neoplastic risk [51]. Moisan et al. [49] demonstrated in a conditional Wtx knockout mouse model the presence of malformations affecting organs derived from mesenchymal progenitors, including kidneys, adipose tissue, heart, spleen and bones, very similar to those reported in children with OS-CS. At the same time, some mice developed bilateral multifocal expansion of renal precursors, but not Wilms tumors, suggesting a complex role of WTX in kidney development, whose alteration can lead to tissue agenesis or overgrowth. Mutations associated to WTX-interacting proteins have also been advocated to explain tumor development [8].

## Discussion and conclusions

OS-CS is a rare bone dysplasia, characterized by longitudinal long bone striations and cranial sclerosis. Typical facial dysmorphism, sensory defects, internal organs anomalies, growth and mental retardation can be associated, helping the physician in the diagnosis, otherwise very challenging in asymptomatic cases. Although rare, because of the severe health impairment that the associated malformations could lead to, and the family inheritance of the disease, OS-CS should be considered in patients affected by or with a family history of deafness, especially if it is associated to facial dysmorphism, sensory defects, internal organ malformations or developmental delay. When OS-CS is suspected, radiographic imaging such as x-rays of the skull and long bones and cranial CT, are required to confirm the presence of the typical bone lesions. Opthalmological examination can be useful to identify optic nerve defects, as for abdominal and heart ultrasound for the detection of internal organs malformations, respectively. Family members should also be investigated. Genetic analyses for *WTX* mutations in the patient and in the patient’s family could be useful for genetic counseling, even if it should be kept in mind that no clear correlation between genotype and phenotype exists.

## Consent

Written informed consent was provided by parents on behalf of the patient for the publication of this case report and any accompanying images.

## Competing interests

The authors declare that they have no competing interests.

## Authors’ contributions

AZ and LP evaluated the patient at the initial expert otolaryngological consultation and follow up visits, and helped in manuscript drafting. LT, FN and AS provided the neurological and genetic consultation to the patient and patient’s mother. DP carried out the genetic studies. GS and NL helped in literature search and manuscript drafting. FG helped in the interpretation of genetic analyses, provided internal medicine consultation to the patient’s mother and performed literature revision and manuscript drafting. MD coordinated the diagnostic and therapeutic process and helped to draft the manuscript. All authors read and approved the final manuscript.

## Supplementary Material

Additional file 1 Clinical, radiological and genetic features of patients diagnosed with OS-CS. Click here for file
