# Supplementary material for: WTX R353X mutation in a family with osteopathia striata and cranial sclerosis (OS-CS): case report and literature review of the disease clinical, genetic and radiological features
Source: Ital J Pediatr. 2012 Jun 20;38:27. doi: 10.1186/1824-7288-38-27 (PMC3416731; doi:10.1186/1824-7288-38-27)
Supplement: Additional file 1 — Clinical, radiological and genetic features of patients diagnosed with OS-CS. [file 1824-7288-38-27-S1.doc]

| **Author, year** | **Gender** | **Defect** | | | | |  | **Mutation** |
| --- | --- | --- | --- | --- | --- | --- | --- | --- |
|  |  | **Head and face** | **Limbs and pelvis** | **Chest and spine** | **Neurologic and developmental** | **Respiratory, Gastrointestinal, Genitourinary, Cardiologic** | **Other** |  |
| **Bar-Oz (1996) [12]** | M (*p*) | MC; BNB; small dysplastic ears | - | - | - | ARDS; PDA; VSD; duodenal web | Died at 4 months of cardiac arrest | - |
|  | F (*pm*) | MC; FB; DPF; BNB  CS; SO; TBS | LSLB | - | - | - | - | - |
| **Bass (1980) [2]** | F | MC; FB; BNB; DPF; PRS; malocclusion; CS | LSLB  FLSIB | - | HL; DD; QI=75 | - | - | - |
| **Berenholz (2002) [1]** | F | CS; CP; FB, BNB; SO | LSLB | - | HL; temporary right facial paralysis | - | - | - |
| **Bloor (1954) [13]**  **Horan (1978) [21]** | F | MC; CS; FB; DPF; HAP; bifid uvula; SO | LSLB  FLSIB | Barrel shaped chest; prominent sternum | - | Nasal obstruction, bronchitis; difficult feeding; distended abdomen, umbilical hernia | - | - |
| **Bueno (1998) [11]** | F (*p*) | MC; CS; FB; dental malocclusion | LSLB | Ribs thickening | HL; spina bifida occulta | - | Short stature | - |
|  | F (*pm*) | MC; FB; CS | LSLB | - | - | - | - | - |
|  | M (*pb*) | MC; FB; CP |  | - | - | Unspecified severe congenital heart disease | Multiple joints luxation |  |
|  | M (*pmgf*) | MC | LSLB | - | - | - | - | - |
| **Clementi (1993) [14]** | F | MC; FB; BNB; telecanthus; narrow palate; dental malocclusion; CS; OS | LSLB | Chest right depression; scoliosis | DD; HL; contraction of visual field | I degree AV block; left ventricular hypertrophy; interventricular defect | Postaxial polydactily, cutaneous IV-Vth toe syndactily | - |
| **Cortina (1981) [15]** | F (*p*) | MC; square head; FB; BNB; CP; obtuse mandible; CS | LSLB | - | - | - | - | - |
|  | F (*pm*) | MC; square head; CS | LSLB  FLSIB | - | - | - | - | - |
| **Currarino (1986) [16]** | M (*p*) | MC; CP; CS | Short fibulas | Cervical kyphosis; lumbar lordosis | HL; speech defects | - | Digital anomalies; short stature | - |
|  | F (*pm*) | Dental defects; CS | - | - | - | - | - | - |
| **De Keyser (1983) [17]** | F (*p*) | MC; FB; BNB; CS | LSLB  FLSIB | Vertebral sclerosis | Gait difficulties; ptosis of left eye; left side internuclear ophtalmoplegia and left abducens paresis; maxillary nerve deficit; HL; numb feeling of left 1^st^ 2^nd^ fingers; spina bifida | Chronic sinusitis; hypertension | Left foot polydactily | - |
|  | F (*pd*) | MC; FB; BNB; CS | LSLB | - | HL | - | - | - |
| **Deniz (2007) [18]** | M | CS | LSLB  FLSIB | Vertebral sclerosis | Lumbar spinal stenosis | - | - | - |
| **Gay (1994) [19]** | F | MC; FB; DPF; CS; OS; telecanthus; mid face hypoplasia, low set ears, HAP; obtuse mandibular angle; delayed dentition | LSLB  FLSIB | Broad, dense clavicles and ribs; scoliosis | HL; 3^rd^ and 12^th^ cranial nerve deficiency; ventricles and large subarachnoid spaces. | ARDS; laryngomalacia; nasal obstruction; ear and respiratory infections | - | - |
| **Holman (2011) [20]**  **Fukuzawa (2010) [44]** | M (p) *ǂ | CS; CP; FB | - | - | - | Cryptorchidism; internal organs matted together; perilobular bilateral nephrogenic rests in kidneys | Reduced bone marrow space | 868insG, G290fs+33X |
| **Holman (2011) [20]** | ? ǂ | MC; FB | - | - | - | - | Syndactily III-IV toes; bilateral club feet | 808delG, V270Cfs+10X |
| **Holman (2011) [20]** | F * | CS | Short lower limbs; bilateral fibula aplasia; Short forearms with bowed radii |  | Dilated lateral ventricles | Pulmonary valve stenosis; ventricular septal defect with overriding aorta; bilateral intralobar nephrogenic rests, nodular nephrogenic blastema; omphalocele | Syndactily of III and IV toe of right foot, II and III toe left foot; II and III fingers left hand | - |
| **Horan (1978) [21]**  **Family 1** | M (*p*) | CS; HAP | LSLB; pelvis sclerosis | Scoliosis; spinal malalignment; vertebral pedicles defects | - | - | - | - |
|  | M (*pb*) | CS; HAP | LSLB; pelvis sclerosis | Scoliosis; spinal malalignment; vertebral pedicles defects | - | - | - | - |
|  | F (*pm*) | CS | LSLB | - | - | - | - | - |
| **Horan (1978) [21]**  **Family 2** | F (*p*) | CS; FB | LSLB; FLSIB | Vertebral sclerosis; pectus excavatum; anterior flaring of lower rib cage; scoliosis | HL; headache | - | - | - |
|  | M (*p half-brother*) | CS; FB | - | - | - | - | - | - |
|  | M (*pf*) | CS; FB | - | - | - | - | - | - |
|  | F (*ppgm*) | CS; FB | LSLB | - | HL | - | - | - |
| **Horan (1978) [21]**  **Family 3** | F (*p*) | CS; FB | LSLB; FLSIB; pelvis sclerosis | Ribs and vertebral sclerosis | HL | - | - | - |
|  | F (*ps*) | CS; FB | LSLB; FLSIB; pelvis sclerosis | Ribs and vertebral sclerosis | HL | - | - | - |
|  | F (*pgm*) | CS; FB | LSLB; FLSIB; pelvis sclerosis | Ribs and vertebral sclerosis | HL | - | - | - |
| **Hurt (1953) [3]** | M | CS | LSLB  FLSIB | Dense ribs; vertical vertebral striation | Mild mental retardation | - | - | - |
| **Jenkins (2009) [8]** | M (*p*)ǂ | CS; micrognathia | Bone sclerosis; bilateral fibula absence, bowing of tibia | - | - | Hypoplastic right ventricle, pulmonary atresia, narrowed pulmonary trunk, right-sided aortic arch, vascular ring; enlarged kidneys; omphalocele | Duplicated distal phalanges digit II, clinodactily IV and V, short I metacarpal digit I; hypoplastic middle phalanges | 671delC ; P224fs+57X |
|  | F (*pm*) | CS | LSLB; bone sclerosis | - | HL | - | - | 671delC ; P224fs+57X |
| **Jenkins (2009) [8]**  **Holman (2011) [20]** | M (*p*) | CS; CP | Bone sclerosis; bilateral fibulae absence | - | Bilateral ventriculomegaly; Arnold Chiari type I | Duplex uretic system on left side of left kidney | - | 780 insA;P260fs+16X |
|  | F (*pm*) | CS | Bone sclerosis; LSLB | - | - | - | - | 780 insA;P260fs+16X |
|  | F (*ps*) | CS; CP | Bone sclerosis | - | - | - | - | 780 insA;P260fs+16X |
| **Jenkins (2009) [8]**  **Holman (2011) [20]** | M | CP | Bone sclerosis; bilateral absent fibula; bilateral bowed tibia, radius and ulna | - | Ventriculomegaly | Omphalocelr | Bilateral hand synpolydactily; duplicated distal phalanges digit II | 1072C>T; R358X |
| **Jenkins (2009) [8]** | M (*p*) | CS | Bone sclerosis | - | DD; HL | Cardiac anomaly | - | 1275C>G; Y425X |
|  | F (*pm*) | CS | Bone sclerosis; LSLB | - | - | - | - | 1275C>G; Y425X |
| **Jenkins (2009) [8]**  **Holman (2011) [20]** | M (*p*) | CS; bifid uvula; telecanthus; epicanthus; FB; BNB; small sella turcica | Bone sclerosis; bilateral hip dysplasia | Increased lumbar lordosis, lumbosacral spondylolisthesis | DD; ventricular dilatation; proximal weakness; hips, knees, ankle contractures | - | - | 1506delA;G502fs+38X |
|  | F (*pm*) | CS | Bone sclerosis; LSLB | - | - | - | - | 1506delA;G502fs+38X |
| **Holman (2011) [20]** | ? * | MC; telecanthus; epicanthus; BNB | - | - | HL | - | Pes calcaneovarus | 1591C>T; R351X |
| **Holman (2011) [20]** | ? | MC; CS; telecanthus; epicanthus; BNB; low set ears | LSLB | Scoliosis | HL; leucomalacia; ventriculomegaly | - | - | 1072C>T;R358X (mosaic) |
| **Jenkins (2009) [8]** | F | CS; CP | Bone sclerosis | - | DD; HL | Cardiac anomaly | - | Gene deletion; no protein |
| **Jenkins (2009) [8]** | F | CS; CP | LSLB; bone sclerosis | - | DD; HL | - | - | Gene deletion; no protein |
| **Jenkins (2009) [8]** | F (*p*) | CS | LSLB; bone sclerosis | - | - | - | - | 502_3delGGinsT; F168fs+1 |
|  | F(*pd*) | CS; CP | LSLB; bone sclerosis | - | - | - | - | 502_3delGGinsT; F168fs+1 |
| **Jenkins (2009) [8]** | F | CS | LSLB; bone sclerosis | - | DD; HL | - | - | 673C>T;Q213X |
| **Jenkins (2009) [8]** | F (p) | CS | Bone sclerosis; LSLB | - | DD | - | - | 867_8delAG;T289fs+33X |
|  | F (pd) | CS | Bone sclerosis; LSLB | - | DD; HL | - | - | 867_8delAG;T289fs+33X |
|  | F pd) | CS; CP | Bone sclerosis; LSLB | - | DD | - | - | 867_8delAG;T289fs+33X |
| **Jenkins (2009) [8]** | F | CS; CP | Bone sclerosis; LSLB | - | HL | Cardiac anomaly | - | 1057C>T;R353X |
| **Jenkins (2009) [8]** | F | CS | Bone sclerosis; LSLB | - | HL | - | - | 1057C>T;R353X |
| **Jenkins (2009) [8]** | F | CS; CP | Bone sclerosis; LSLB | - | DD | Cardiac anomaly | - | 1072C>T; R358X |
| **Jenkins (2009) [8]** | F | CS | Bone sclerosis; LSLB | - | HL | - | - | 1072C>T; R358X |
| **Jenkins (2009) [8]** | F | CS; CP | Bone sclerosis; LSLB | - | DD | - | - | 1072C>T; R358X |
| **Jenkins (2009) [8]** | F | CS; CP | Bone sclerosis; LSLB | - | DD; HL | Cardiac anomaly | - | 1072C>T; R358X |
| **Jenkins (2009) [8]** | F | CS | Bone sclerosis; LSLB |  | HL |  |  | 1637insT;F545fs+34X |
| **Jones (1968) [22]** | F | CS; CP | LSLB; FLSIB | Vertebral and ribs sclerosis | HL | - | - | - |
| **Joseph (2010) [40]**  **Lazar (1999) [10]** | M | MC; telecanthus; FNB;CP;micrognathia; ankylosis of temporomandibular joint | LSLB; FLSIB | - | HL; intellectual impairment; enlarged ventricles | - | Club feet | 1108G>T; E370X Mosaicism |
| **Kondoh (2001) [42]** | F | TPF; FB; BNB; CP; narrow forehead; mouth granuloma; accessory ear; micrognathia; low set, deformed ears; short lingual frenulum | LSLB; FLSIB | Malalignment of cervical spine | DD; hypotonia; impingement of spinal cord (narrow spinal canal); mental retardation | - | Short stature | - |
| **Koudstaal (2008) [23]** | F | MC; CS; CP; telecanthus; open bite; BNB; malocclusion; narrow maxilla; late dental development | - | - | HL | Pulmonary hypertension | Refractory eye disorder | - |
|  | F | MC; CS; CP; FB; telecanthus; open bite; BNB; malocclusion; narrow maxilla; late dental development | - | - | HL | Pylorostenosis | Refractory eye disorder | - |
| **Kornreich (1988) [41]** | F | CP; FB; square skull; BNB | - | - | Right facial nerve palsy | Ventricular septal defect; recurrent otitis media | - | - |
| **Lee (2004) [24]** | F | CS | LSLB; FLSIB | - | - | - | - | - |
| **Lüerβen (2006) [37]** | M | MC; CP; BNB; telecanthus; askent nose, small nares | LSLB; FLSIB |  | Left diaphragm paralysis; expansion of cerebral ventricles with; HL | Recurrent ear infections; multiple heart defects in the septum; spongiosa myocardia; insufficient tricuspid valve | - | - |
| **Magliulo (2007) [6]**  **Present case** | F (*p*) | CS; BNB; FB; telecanthus; epicanthus; hypoplastic maxillae; delayed dentition; low set ears; HAP; | LSLB | Pectus excavatum | HL | Recurrent otitis media | - | c.1057C>T; p.R353X |
|  | F (*pm*) | Hypoplastic maxillae; dental-position abnormalities; BNB | LSLB | - | - | - | - | c.1057C>T; p.R353X |
| **Mohan (1990) [25]** | M | CS | LSLB; FLSIB | Longitudinal striations, widened ribs | - | - | - | - |
| **Nakamura (1985) [26]** | M (*p*) | CP; CS; mandible sclerosis and striations | LSLB | Ribs sclerosis | - | - | - | - |
|  | F (*ps*) | Mandible sclerosis and striations | LSLB | - | - | - | - | - |
|  | F (*pm*) | CS; mandible sclerosis and striations | LSLB | - | - | - | - | - |
| **Nakamura (1998) [4]** | M | CS; SO | LSLB; FLSIB | Thick, striated ribs, vertebrae and clavicles | - | - | Short stature | - |
| **Odrezin (1992) [27]** | F | MC; CS; FB; micrognathia; partial hypodontia, telecanthus | - | - | HL | Ventricular septal defect | - | - |
| **Paling (1981) [28]** | F | CS; MC; FB; BNB; DPF; HAP; dental abnormalities | LSLB; valgus elbows and knees; FLSIB | Keel-shaped sternum;  thick ribs; lumbar lordosis; asymmetric rib cage | DD; HL | Nasal obstruction, rhinorrhea | High arched feet; toe dorsiflexion deformity | - |
| **Pellegrino (1997) [29]** | M (*p*) | MC; FB; CP; bitemporal narrowing; occipital prominence; epicanthus; telecanthus; small, low set ears; BNB; micrognathia | LSLB | Broad, flat ribs; osteosclerosis; mild thoracolumbar gibbus | Partial agenesis of corpus callosum; seixures; hydrocephalus, cortical atrophy | Intestinal malrotation | Bilateral clinodactily of V finger |  |
|  | M (*p half brother*) | MC; FB; abnormal left ear; webbed neck | LSLB | Bilateral II finger camptodactily; clubbed feet; bell shaped thorax; diffuse osteosclerosis | Hypotonia; seizures | Omphalocele; malrotation with gastric volvulus; right multicystic kidney; atrial septal, ventriculoseptal defect; PDA; tricuspid insufficiency | Died at 3 months of heart failure |  |
|  | M (*pb*) | MC; FB; telecanthus; low set ears; micrognathia; CP; bifid uvula and epiglottis; hypoplastic maxilla | LSLB; absent fibulae | Abnormal 7^th^ rib; osteosclerosis; | Increased ventricles size; cortical atrophy | Incomplete rotation (left liver, right small bowel); atrial and ventriculoseptal defects; micropenis; undescended left testicle | Died at 2.5 yr of an aspiration |  |
|  | F (*ps*) | MC; CS; FB; telecanthus; low set, posteriorly angulated ears; CP; bifid uvula and epiglottis | - | - | - | Intestinal malrotation | Broad thumbs; clinodactyly of I toe  Died of aspiration at 4 days | - |
|  | M (*pb*) | MC; CS; FB; telecanthus; low set, posteriorly angulated ears | Osteosclerosis of long and iliac bones | - | - | - | - | - |
|  | F (*pm*) | MC; CS | LSLB | - | - | - | - | - |
| **Perdu (2010) [30]**  **Keymolen (1997) [38]**  **Holman (2011) [20]**  **Family 1** | F (*p*) | MC; CS; telecanthus | LSLB | - | Hydrocephalus; hypoesthesia on the palmar side on the I and II left fingers; right leg paresis; palpebral ptosis; spina bifida occulta; bilateral HL | Chronic sinusitis | Polydactily | 1267delC;L423fs+25X |
|  | F (*pd*) | MC; CS; BNB; FB; telecanthus; irregular, malpositioned upper incisors | LSLB | - | HL | Recurrent otitis | - | 1267delC;L423fs+25X |
|  | M (*p maternal cousin*) | MC; CS; CP; FB; telecanthus; epicanthus; HAP; small mouth | - | - | Delayed speech; HL | Severe stomach problems | - | 1267delC;L423fs+25X |
| **Perdu (2010) [30]**  **Family 2** | F | CS; CP; FB; dolichocephaly; deep-set, small eyes; epicanthus; telecanthus; BNB; low set ears; HAP | LSLB | Thoracolumbar gibbus; narrow and sclerotic pelvic bones | HL; ventriculomegaly, widening of basal cisterns and interemispheric fissure, megacisterna magna; mild psychomotor developmental delay; recurrent headaches; bilateral knee contracture | Intestinal malrotation; bicornate uterus | - | 337delG;G113fs+58X |
| **Perdu (2010) [30]**  **Family 3**  **Savarayan (1997) [36]** | F (*p*) | MC; CS; CP; FB | LSLB; proximal osteolysis of the fibulae | - | HL | Atrial and ventricular septal defect; anterior ectopic anus; left hydronephrosis with dilated left ureter; small right kidney | Cutaneous syndactyly of right III and IV fingers | Deletion; no protein |
|  | F (*pm*) | MC; CS | LSLB | - | - | - | - | Deletion; no protein |
|  | F (*pmgm*) | MC; CS; CP; FB; midface hypoplasia | LSLB | - | HL | - | Short stature | Deletion; no protein |
|  | F (ps) | CS; CP; nose partial absence | - | - | Alobar holoprosencaphaly; absence of olfactory nerves | - | - | Abortion |
|  | F (*pmggm*) | CS | - | - | - | - | - | - |
| **Perdu (2010) [30]**  **Family 4** | F (*p*) | CS; FB; bitemporal narrowing; tooth malpositioning; hypotelorism | LSLB | - | Headache; HL | Recurrent otitis media | Myopia | 1072 C>T;R358X |
|  | F (*pm*) | FB; bitemporal narrowing; tooth malpositioning; hypotelorism, | - | - | HL | - | - | - |
| **Perdu (2010) [30]**  **Family 5** | F | MC; facial asymmetry (prominent left side); FB; epicanthus; BNB | LSLB | - | HL | - | Short stature | 1072 C>T;R358X |
| **Perdu (2010) [30]**  **Family 6** | F | MC; CS; CP; FB; deep set eyes; telecanthus; BNB | LSLB | Widened ribs | Hypotonia; HL; DD | Ventral septal defect; PDA | Short stature | 1072 C>T;R358X |
| **Perdu (2010) [30]**  **Family 7** | F | MC; CS; FB; low-set and posteriorly rotated ears; telecanthus; epicanthus; BNB; HAP; crowded teeth; microretrognathia; | LSLB | - | DD; QI=77; corpus callosum partial agenesis; HL; optic foramen stenosis | Laryngotracheomalacia; absence of laryngeal cartilages; thickened epiglottis | Short stature | 1072 C>T;R358X |
| **Perdu (2010) [30]**  **Konig (1996) [43]**  **Holman (2011) [20]**  **Family 8** | F (*p*) | MC; CS | LSLB | - | - | - | - | 811C>T; Q271X |
|  | F (*pd*) | MC; CS; CP; telecanthus; left maxillary sinus hypoplasia | LSLB | Pectus excavatum | HL | Mitral valve insufficiency/stenosis | Nasal speech | 811C>T; Q271X |
|  | F (*pgd*) | MC; CS; BNB; telecanthus; maxillary hypoplasia; prominent mandible; low-set posteriorly angulated ears | LSLB | Striations of ribs and scapulae; long, straight clavicles; wide ribs | Hydrocephalus; right facial palsy | Atrial septal defect; PDA | - | 811C>T; Q271X |
|  | F (*pgd*) | MC; CS; flat face; BNB | LSLB | - | - | - | - | 811C>T; Q271X |
|  | F (*pggd*) | MC; CS | - | - | Moderate speech retardation | - | - | 811C>T; Q271X |
|  | M (*pggs*) | MC; CS; PRS; FB; dolicocephaly; telecanthus; dental malformations; deep, posteriorly rotated ears | Dense metaphyses of tibia and femur | Abnormally long clavicles with broad ends | HL | Ventricular dilatation | - | 811C>T; Q271X |
|  | M (*pggs*) | MC; CS; CP; FB; epicanthus; telecanthus; BNB; full lips; omega epiglottis | Dense metaphyses of tibia and femur; hip dysplasia | Abnormally long clavicles with broad ends | - | - | Sleep apnea | 811C>T; Q271X |
| **Perdu (2011) [31]**  **Family 1** | M (*p*) | MC; upturned nasal tip; long philtrum; micro- and retrognathia; low set, posteriorly rotated ears; underdeveloped tongue | Bilateral fibula aplasia | - | - | Hypoplastic left heart; intestinal malrotation; | Died soon after birth | 654delG; E216fs+62X |
|  | F (*pm*) | MC; CS; telecanthus; flat face; HAP; BNB | LSLB | - | HL; difficulties at school | - | - | 654delG; E216fs+62X |
| **Perdu (2011) [31]**  **Family 2** | M (*p*) | CS; dolicocephaly; telecanthus; DPF; eyelids ectropion; BNB; CP; dysplastic low set ears; short neck; hypodontia; dysplastic teeth | LSLB; bilateral proximal fibular hypoplasia | Scoliosis; short and broad clavicles | Corpus callosum hypoplasia; ventricular dilatation; HL; DD; general seizures; sleep apnea, Arnold Chiari I; selective mutism; IQ 65 | Umbilical hernia; hypoplastic abdominal muscles; cardiac septal defect II, PDA | Broad thumb and halluces; Flexion contractures of the fingers II-V; growth reatardation | 429T>A; C143X |
|  | F (*pm*) | MC; CS; long face; long philtrum; thin lips; HAP | LSLB; mild legs asymmetry | Scoliosis | - | - | - | 429T>A; C143X (heterozygous) |
| **Robinow (1984) [32]** | F | MC; CS; FB; telecanthus; BNB; bilateral epicanthus | LSLB  FLSIB | Thick ribs | Enlarged verntricles and subarachnoideal spaces | Stenotic perineal anus | - | - |
| **Rott (2003) [33]** | M (*p*) | CS; FB; dysplastic, low set ears; telecanthus; BNB; CP; small tongue | - | - | Cerebellar vermis hypoplasia; large hemispheres; dilated asymmetric ventricles, microgyria; | Imperforate anus; blind ending colon; patent ductus arteriosus, open foramen ovale; cyptorchidism | Duplication of distal phalanx of I and II right digits; bilateral cutaneous syndactyly of III and IV, II and III toes; club feet; hypothyreosis | - |
|  | F (*pm*) | CS; FB; telecanthus; epicanthus; BNB | LSLB | - | HL | - | - | - |
|  | F (*pm’s aunt*) | CS | LSLB | - | HL | - | - | - |
|  | F (*pmgm*) | CS; FB; telecanthus; epicanthus; BNB | LSLB | - | HL | - | - | Random X inactivation (repeat numbers of 218 and 221 bp) |
| **Schnyder (1980) [34]** | M | MC; CS; SO | LSLB; FLSIB | Striation of ribs and vertebrae | - | - | - | - |
| **Viot (2002) [35]**  **Family 1** | F | CS; PRS; irregular teeth | LSLB | Scoliosis | DD; corpus callosum hypoplasia; hydrocephalus | Cardiac defect | - | - |
| **Viot (2002) [35]**  **Jenkins (2009) [8]**  **Holman (2011) [20]**  **Family 2** | M (*p*) | CS; irregular teeth; choanal hypoplasia; telechantus; FB | LSLB | - | DD; HL; corpus callosum hypoplasia; cerebral ventricular dilatation | Cardiac defect; Hirschprung disease | Valgus feet | - |
|  | F (*pm*) | CS | LSLB | - | - | - | - | - |
| **Viot (2002) [35]**  **Family 3** | F | CS | LSLB | - | DD; cerebral ventricular dilatation | Laryngotracheomalacia | - | - |
| **Viot (2002) [35]**  **Family 4** | F (*p*) | CS; CP; PRS | LSLB | - | - | - | - | - |
|  | F (*pm*) | CS | LSLB | - | Cerebral ventricular dilatation; cranial nerves palsy | - | - | - |
| **Viot (2002) [35]**  **Family 5** | F | CS; CP | - | Scoliosis | DD; HL | - | - | Died at 20 months |

**LEGEND TO TABLE:** ARDS= acute respiratory distress syndrome; AV= atrio-ventricular; BNB= broad nasal bridge; CP= cleft palate; CS= cranial sclerosis; DD= developmental delay; DPF= downslating palpebral fissures; F= female; FB= frontal bossing; FLSIB= fan like striations of iliac bones; HAP= high arched palate; HL= hearing loss; LSLB= longitudinal striations of long bones; M= male; MC= macrocephaly; p = proband; pb= proband brother; pd= proband daughter; PDA= patent ductus arteriosus; pf= proband father; pgd= proband grand-daughter; pggd= proband grand granddaughter; pggs= proband grand grandson; pm= proband mother; pmgf= proband maternal grandfather; pmggm= proband maternal great grandmother; ppgf= proband paternal grandfather; PRS= Pierre Robin Syndrome; ps= proband sister; SO= sinuses obliteration; TBS= thickening of the base of the skull; VSD= ventricular septal defect.

* Affected siblings for whom specific alterations have not been reported; ǂ = lethally affected male relatives
